# Supplementary figures and images for: Recurrent promoter mutations in melanoma are defined by an extended context-specific mutational signature
Source: PLoS Genet. 2017 May 10;13(5):e1006773. doi: 10.1371/journal.pgen.1006773 (PMC5443578; doi:10.1371/journal.pgen.1006773)

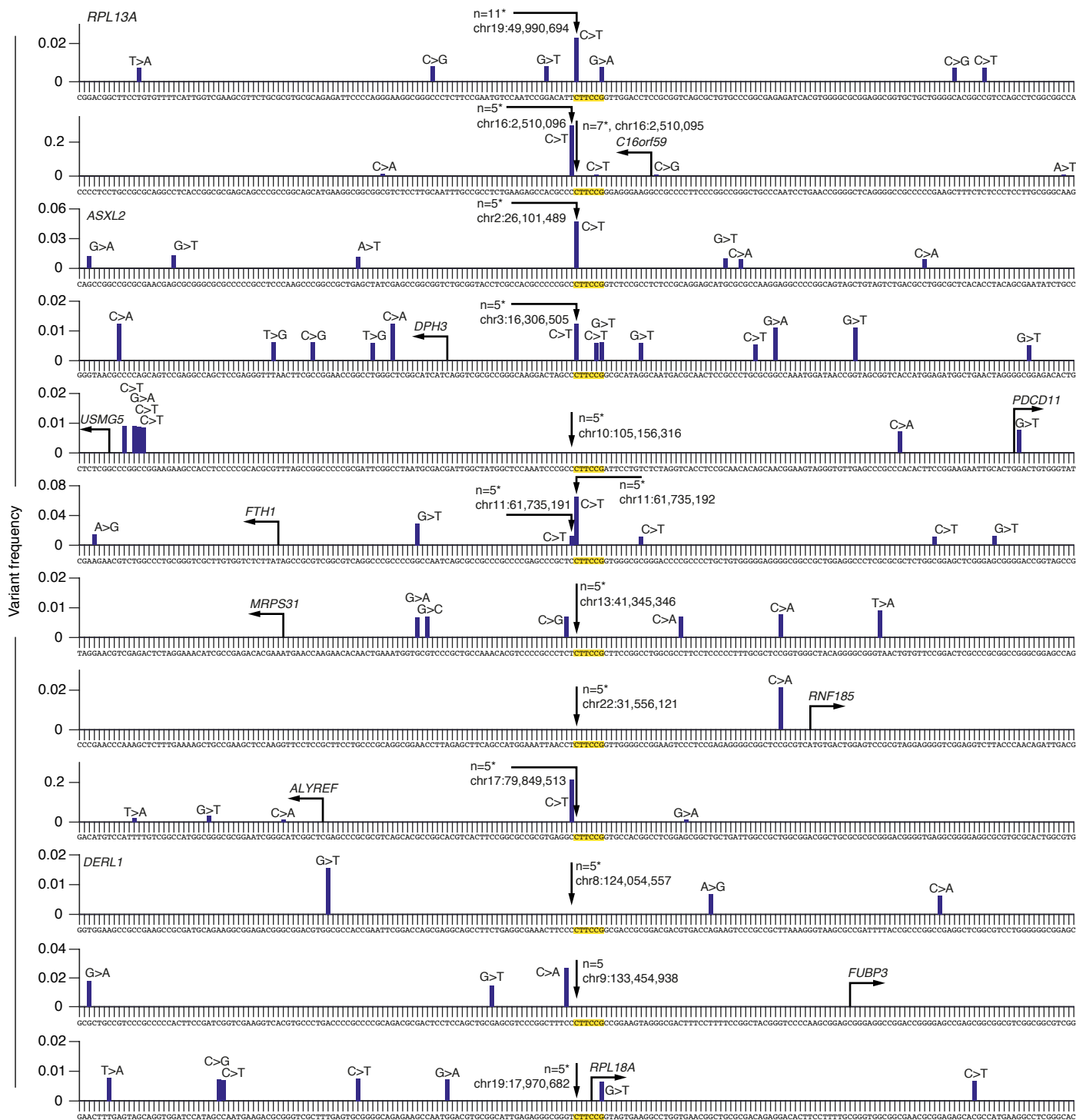

Supplement: S1 Fig — Recurrent CTTCCG-related promoter hotspot sites identified in melanoma (mutated in > = 5/38 TCGA tumors) were examined for mutations in a sample of sun-exposed normal skin. The graphs show variant allele frequencies for mutations in genomic regions centered on these sites, based on whole genome sequencing data from sun-exposed normal eyelid skin obtained from Martincorena et al.[19]. Known population variants were excluded, but all other deviations from the reference sequence are shown regardless of allele frequency. (PDF) [file pgen.1006773.s001.pdf]

**a**

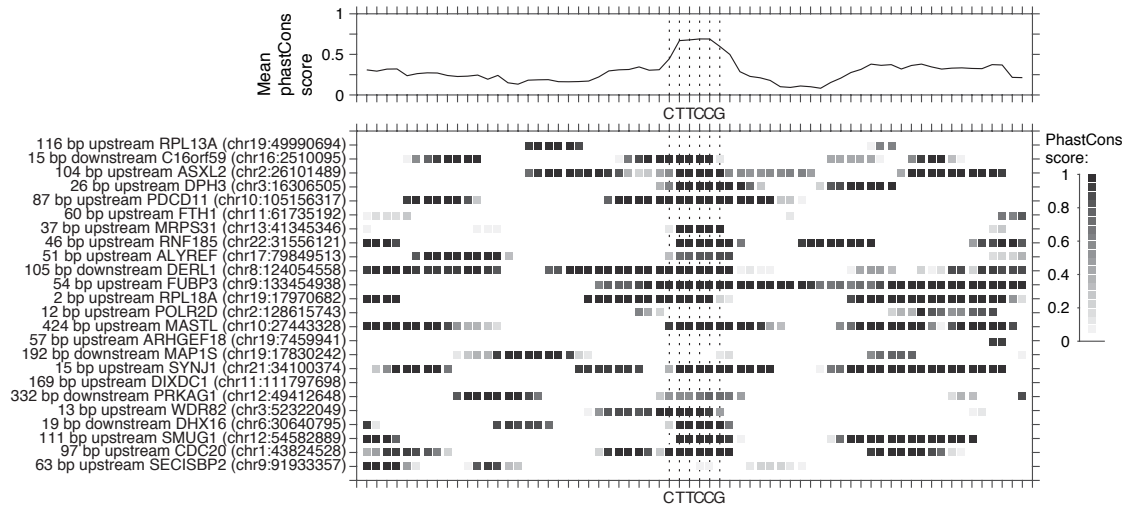

**b**

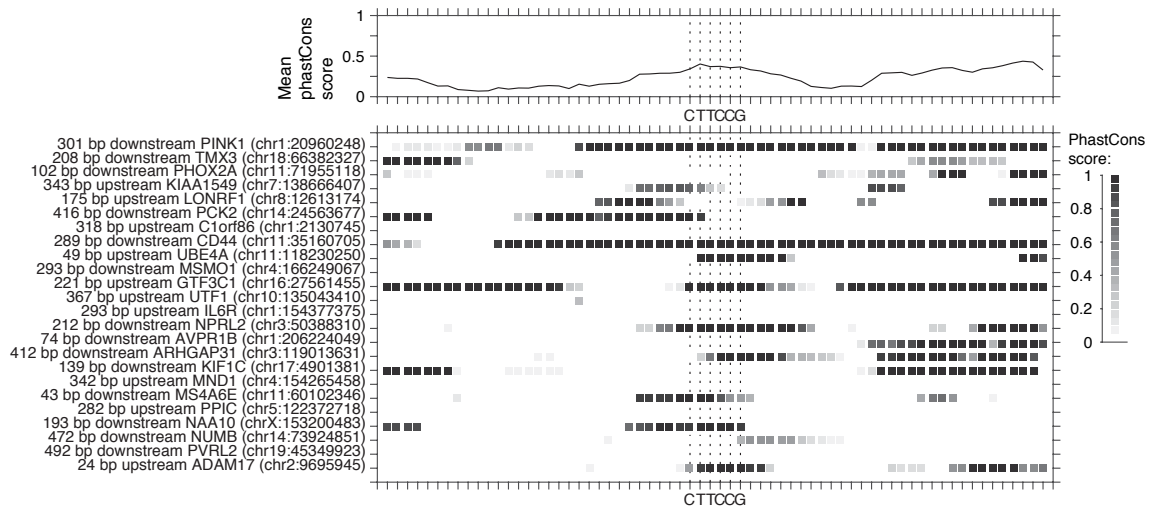

Supplement: S2 Fig — PhastCons conservation scores at CTTCCG sites in melanoma promoter hotspot sites (a) and in 24 randomly chosen CTTCCG sites less than 500 bp from TSS of highly expressed genes, that were not mutated in any tumor (b). PhastCons conservation scores were derived from multiple alignments of 100 vertebrate species and downloaded from the UCSC genome browser. (PDF) [file pgen.1006773.s002.pdf]

**a**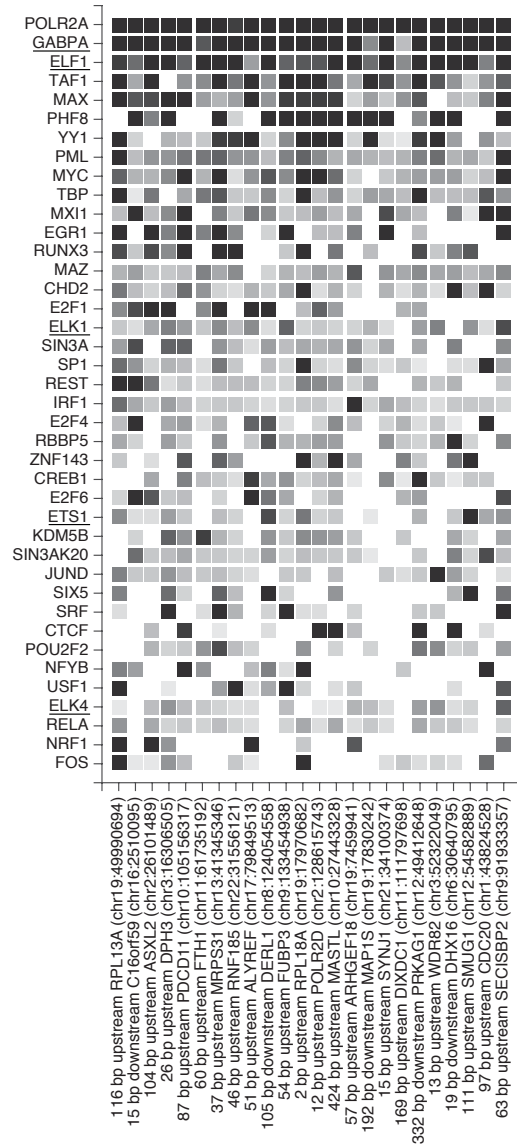**b**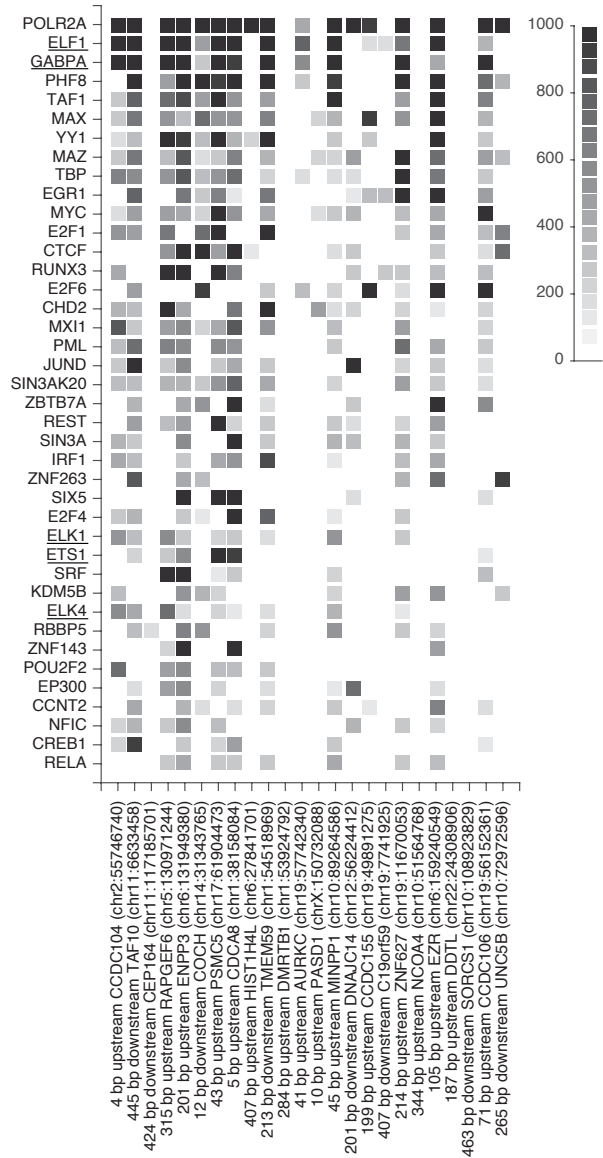

Supplement: S3 Fig — Normalized scores for ChIP-seq peaks from 161 transcription factors in 91 cell types at NCTTCCGN sites (ENCODE track wgEncodeRegTfbsClusteredV3 obtained from the UCSC genome browser). (a) Promoter mutation hotspot sites. (b) 24 randomly chosen NCTTCCGN sites less than 500 bp from TSS of highly expressed genes that were not mutated in any tumor. In both panels, factors are ranked by mean signal across the 24 sites, with the 40 top factors being shown. Transcription factors from the ETS transcription factor family are underlined. The given genomic position for each site, indicated in the x-axis labels, is the location of the motif CTTCCG. (PDF) [file pgen.1006773.s003.pdf]
