# Supplementary material for: Recurrent promoter mutations in melanoma are defined by an extended context-specific mutational signature
Source: PLoS Genet. 2017 May 10;13(5):e1006773. doi: 10.1371/journal.pgen.1006773 (PMC5443578; doi:10.1371/journal.pgen.1006773)
Supplement: S1 Table — The table complements Fig 1a and shows sites with a lower degree of mutation recurrence (3/38 melanomas, 8%), but is otherwise identical to Fig 1a. Approximately 50% of sites at this level of recurrence conform to the CTTCCG pattern. (PDF) [file pgen.1006773.s004.pdf]

Rec[a]	Chr[b]	Position	Ref[c]	Var[d]	Sequence context[e]	Dist[f]	Gene[g]	Expr. tier[h]	*P*[i]	Dist[j]	Gene[k]	Expr.tier[l]	*P*[m]
3	6	24721423	C	T	CCCGCCACTCCTTCCGCCCCC	-359	*C6orf62*	3	0.13				
3	12	498776	C	T	GTGACGCTTTCTTCCGGCGCG	-156	*KDM5A*	3	0.787	263	*CCDC77*	3	0.0513
3	9	131038413	C	T	GCCACGCCCCCTTCCGCTTCA	-139	*GOLGA2*	3	0.871				
3	1	25559064	C	T	AGCCCCGCCCCTTCCGGGAGG	-80	*SYF2*	3	0.552				
3	2	73964607	C	T	CCCGCCCATTCTTCCGCCTCC	-80	*TPRKB*	3	1				
3	22	35795975	C	T	ACCTCGCCTCCTTCCGGGCTC	-80	*MCM5*	3	0.234				
3	19	1812349	C	T	CCCCCGGCCCCCTTCCGGGGT	-74	*ATP8B3*	2	0.516				
3	6	31940123	C	T	AAAATAGGGTCTTCCGGCGCA	-54	*DOM3Z*	3	0.588				
3	14	50779320	C	T	CGGCTTCTTTCTTCCGGCTCG	-54	*L2HGDH*	2	0.588	274	*ATP5S*	2	0.482
3	4	2936631	C	T	ACGTTCTCTTCCGGCCGGAGT	-45	*MFSD10*	3	0.0832				
3	1	100598553	C	T	CCATCGGATTCTTCCGGTTCT	-42	*SASS6*	2	0.588	-152	*TRMT13*	2	0.0513
3	3	101280671	C	T	CCGCCCTCTCCCTTCCGGCGC	-34	*TRMT10C*	3	0.482				
3	11	1330918	C	T	GGCACCGCCCCTTCCGGCTCT	-34	*TOLLIP*	3	0.279				
3	22	43011002	C	T	CGTCCCCGCCCCTTCCGGTTC	-34	*POLDIP3*	3	0.516				
3	2	70056751	C	T	TTGCCCCGCCCCTTCCGGAGG	-22	*GMCL1*	2	0.957				
3	13	29233226	C	T	GGACGCACTTCCGGCGGATGT	-14	*POMP*	3	0.745				
3	10	7830002	C	T	GCCCCACCTCCTTCCGCCTCT	-12	*KIN*	2	0.871	-89	*ATP5C1*	2	0.588
3	2	198318145	C	T	CCCCTTCTTCCCTTCCGGGTT	-1	*COQ10B*	3	0.417				
3	7	23338823	C	T	CCAAGTAGCTCTTCCGGGTCA	5	*MALSU1*	2	0.213				
3	6	170893742	C	A/T	CGCCTCTTGCCTTCCGGCCCG	6	*PDCD2*	3	0.871				
3	13	41837733	C	T	TGGTTCACTTCTTCCGGGTTA	9	*MTRF1*	2	0.626				
3	11	46958262	C	T	TCCCGTCCCCCTTCCGCCGCG	15	*C11orf49*	3	1				
3	20	57607411	C	T	CGCCCCGCCTCTTCCGCTTCT	26	*ATP5E*	3	0.588				
3	X	153059915	C	T	ATTCACGCCCCCTTCCGGCGC	63	*IDH3G*	3	0.256				
3	16	83841526	C	T	CCTCGAGGCCCTTCCGGTGCG	79	*HSBP1*	3	0.665				
3	8	124054558	C	T	GAAACTTCCCCTTCCGGCGAC	105	*DERL1*	3	0.914	351	*WDR67*	3	1
3	14	20585071	C	T	CTGTGTTTTTCCTCCTTATCT	-494	*OR4K17*	1	-[o]				
3	5	137800769	C	T	GGCGGGGGATCCTTCCTGCTC	-409	*EGR1*	3	0.705				
3	3	12883297	C	T	GAAGTAAATTCCTCCCTCCAC	-210	*RPL32*	3	0.914				
3	3	122135048	C	T	ATGTTCATTTCCGGTCTTTTT	-166	*WDR5B*	2	0.787				
3	11	47448149	C	T	TTCTCCCTCCCTTTCCGGTTT	-156	*PSMC3*	3	0.957				
3	2	65283371	C	T	CCCCCTACTTCGTCCTGGCCT	-134	*CEP68*	2	0.588				
3	8	67579583	C	T	ACTTGTAGTTCCTTCTGGACT	-131	*VCPIP1*	2	0.482	-267	*SGKL*	2	0.871
3	19	54641318	C	T	TGCCCCCTTTCGCGGATTGGG	-125	*CNOT3*	3	0.705				
3	16	68119138	C	T	CACGTGACTTCCTTCCTTCTC	-108	*NFATC3*	2	0.279				
3	1	38478324	C	T	AGCCGGCTTTCAGGAAGTACG	-89	*UTP11L*	3	0.279				
3	8	57124164	C	T	GCCCACTCCTCCCGCCTCGGC	-80	*CHCHD7*	3	0.745	-305	*PLAG1*	3	0.304
3	8	56987141	C	T	CAGGAAATATCCCGGGCCCTA	-72	*RPS20*	3	0.213				
3	15	90931383	C	T	GGCCCCGCCTCTTTCCGGCCG	-66	*IQGAP1*	3	0.159				
3	19	48867542	C	T	CCCCCTCCCTCTTTCCGCCTA	-48	*TMEM143*	2	0.665	-109	*SYNGR4*	2	0.664
3	19	48248748	C	T	GCGCAGATTTCCCACCCTCTT	-30	*GLTSCR2*	3	0.116				
3	2	234763235	C	T	TCCCTGCCTTCCTCTCGGTTT	-23	*HJURP*	2	0.957				
3	1	234509179	C	T	CTTCCTGTTTCGCTTTTATCT	-22	*COA6*	3	0.516				
3	16	2510073	C	T	AAGGCCGCCCCTTCCCGGCCG	-7	*C16orf59*	2	0.705				
3	14	55658403	C	T	ATTCAAATATCGCACGGAGCA	-7	*DLGAP5*	2	0.829				
3	19	36870099	C	T	GCTCGCAGTTCTTTCCGGCTT	2	*ZFP14*	2	0.256				
3	12	100660920	C	T	GACGTCACTTCCTGCCGGTTC	3	*SCYL2*	3	0.417	-63	*DEPDC4*	3	0.705
3	14	31028336	C	T	GCCGACCGCTCTTTCCGGGTT	8	*G2E3*	2	0.552				
3	6	34855824	C	T	GATCTTACTTCCTGTCGTCGC	42	*TAF11*	3	0.957				
3	15	40675107	C	T	GAGTGCGATTCCCACCCACTT	186	*KNSTRN*	3	0.829				
3	1	242011463	C	T	GACGTCACATCCTCTGGGCGG	195	*EXO1*	2	0.914				
