# Supplementary material for: Recurrent promoter mutations in melanoma are defined by an extended context-specific mutational signature
Source: PLoS Genet. 2017 May 10;13(5):e1006773. doi: 10.1371/journal.pgen.1006773 (PMC5443578; doi:10.1371/journal.pgen.1006773)
Supplement: S2 Table — aMutation frequency (fraction of tumors having a mutation) in the original analysis based on 38 TCGA tumors, as shown also in Fig 1a. bMutation frequencies for these sites across 25 melanoma tumors as reported by Berger et al. [17]. c0.08 was previously obtained using a different mutation calling pipeline applied to the same data[10] while 0.04 refers to the calls provided by Berger et al. See main Fig 1a for an explanation of remaining columns. (PDF) [file pgen.1006773.s005.pdf]

Rec	Chr	Pos	Ref	Var	Context	Dist	Gene	Freq[a]	Berger freq.[b]
11	19	49990694	C	T	TCCGGACATTCTTCCGGTTGG	-116	*RPL13A*	0,29	0,12
10	5	1295250	C	T	CCCGACCCCTCCCGGGTCCCC	-88	*TERT*	0,26	0,48
7	16	2510095	C	T	AGCCACGCCCCTTCCGGGAGG	15	*C16orf59*	0,18	0,12
7	5	1295228	C	T	GCCCAGCCCCCTCCGGGCCCT	-66	*TERT*	0,18	0,2
5	2	26101489	C	T	CGCCCCCGCCCTTCCGGTCTC	-104	*ASXL2*	0,13	0,04
5	10	105156316	C	T	CAAATCCCGCCCTTCCGATTC	-88	*PDCD11*	0,13	0,08
5	11	61735192	C	T	GAGCCCGCTCCTTCCGGTGGG	-60	*FTH1*	0,13	0,08
5	11	61735191	C	T	CGAGCCCGCTCCTTCCGGTGG	-59	*FTH1*	0,13	0,04
5	9	133454938	C	T/+T	CCGGCTTTCCCTTCCGCCGGA	-54	*FUBP3*	0,13	0
5	17	79849513	C	T	CGCGTGAGGCCTTCCGGTGCC	-51	*ALYREF*	0,13	0,04
5	22	31556121	C	T	AAATTAACCTCTTCCGGTTGG	-46	*RNF185*	0,13	0,08
5	13	41345346	C	T	CCCGCCCTCTCTTCCGCTTCC	-37	*MRPS31*	0,13	0
5	3	16306505	C	A/T/G	AGGACTAGCCCTTCCGGCGCA	-26	*DPH3*	0,13	0,04[c]
5	19	17970682	C	T	GAGGGCGGGTCTTCCGGTAGT	-2	*RPL18A*	0,13	0,12
5	16	2510096	C	T	GAGCCACGCCCCTTCCGGGAG	16	*C16orf59*	0,13	0,08
5	8	124054557	C	T	CGAAACTTCCCCTTCCGGCGA	106	*DERL1*	0,13	0
5	5	1295242	C	T	CTCCCGGGTCCCCGGCCCAGC	-80	*TERT*	0,13	0
4	10	27443328	C	T	AGCGCCTCGCCTTCCGGGGCG	-424	*MASTL*	0,11	0,04
4	11	111797698	C	T	GTAGACAGGCCTTCCGGCCCC	-169	*DIXDC1*	0,11	0
4	12	54582890	C	T	ATTTAGTGCGCCTTCCGGGAT	-112	*SMUG1*	0,11	0
4	12	54582889	C	T	TTTAGTGCGCCTTCCGGGATT	-111	*SMUG1*	0,11	0,08
4	1	43824529	C	T	AGGGGGCGGGCCTTCCGGGGA	-96	*CDC20*	0,11	0,08
4	9	91933357	C	T	CCCGCCCTTTCTTCCGGCCGG	-63	*SECISBP2*	0,11	0
4	19	7459940	C	T	GGGCACGCCTCCTTCCGGGTC	-58	*ARHGEF18*	0,11	0,08
4	19	7459941	C	T	GGCACGCCTCCTTCCGGGTCA	-57	*ARHGEF18*	0,11	0,08
4	3	52322052	C	T	GACGTCACTTCCGGCCCCCTA	-16	*WDR82*	0,11	0
4	21	34100374	C	T	CGGGGCGGATCTTCCGCCCCC	-15	*SYNJ1*	0,11	0,04
4	2	128615744	C	T	AGACCACGCCCCTTCCGCGGC	-13	*POLR2D*	0,11	0,04
4	6	30640796	C	T	AAGTACAGCCCCTTCCGGGCT	18	*DHX16*	0,11	0
4	19	17830242	C	T	GTCTTCAGCCCTTCCGGTGCG	192	*MAP1S*	0,11	0
4	12	49412648	C	T	GGTTCCTTGCCTTCCGCCCCA	332	*PRKAG1*	0,11	0
4	19	2151793	C	T	ACTCCGCCTTCTTCCTAGTTC	-228	*AP3D1*	0,11	0
