# Supplementary material for: Recurrent promoter mutations in melanoma are defined by an extended context-specific mutational signature
Source: PLoS Genet. 2017 May 10;13(5):e1006773. doi: 10.1371/journal.pgen.1006773 (PMC5443578; doi:10.1371/journal.pgen.1006773)
Supplement: S3 Table — Melanoma hotspot positions were investigated in 8 cSCC tumors[37]. In cases where mutations are present, the variant allele frequency is shown for each individual sample (columns) and site (rows), with variant frequencies below 0.2 given within parentheses. aMutation frequency across the 8 cSCC tumors[37], only considering mutations with a variant frequency of at least 0.2. bMutation frequency across the 38 TCGA melanoma tumors. cTotal number of called mutations as reported by Zheng et al. [18]. dNumber of promoter hotspot mutations with variant frequency of at least 0.2. eNumber of deleterious mutations in SCC driver genes with a variant frequency of at least 0.2. Non-synonymous mutations that were considered deleterious by PROVEAN[38] or damaging by SIFT[39] were counted as driver mutations. (PDF) [file pgen.1006773.s006.pdf]

Sample	WT9	WT11	WT12	WT10	WT13	WT8	WT6	WT7	Total mut. freq. [a]	TCGA SKCM mut. freq. [b]
**RPL13A chr19:49990694**	(0.19)	(0.083)	-	-	0.33	0.54	0.47	(0.051)	0.38	0.29
**C16orf59 chr16:2510095**	(0.08)	-	-	-	-	-	-	-	0	0.18
**ASXL2 chr2:26101489**	-	-	-	0.62	-	-	0.32	(0.16)	0.25	0.13
**PDCD11 chr10:105156316**	0.36	-	-	-	-	-	-	0.46	0.25	0.13
**FTH1 chr11:61735192**	-	-	1	-	0.43	-	-	0.41	0.38	0.13
**FTH1 chr11:61735191**	(0.059)	0.75	0.67	-	-	0.7	(0.12)	0.33	0.5	0.13
**FUBP3 chr9:133454938**	-	-	-	-	-	-	-	-	0	0.13
**ALYREF chr17:79849513**	-	0.21	-	0.41	-	-	-	0.28	0.38	0.13
**RNF185 chr22:31556121**	-	-	-	-	-	-	0.39	-	0.12	0.13
**MRPS31 chr13:41345346**	-	-	-	-	-	-	-	-	0	0.13
**DPH3 chr3:16306505**	-	-	-	0.25	-	(0.16)	0.57	-	0.25	0.13
**RPL18A chr19:17970682**	-	(0.14)	-	-	-	-	-	-	0	0.13
**C16orf59 chr16:2510096**	-	-	-	(0.025)	0.45	-	-	(0.054)	0.12	0.13
**DERL1 chr8:124054557**	-	-	-	0.23	-	-	-	-	0.12	0.13
**MASTL chr10:27443328**	-	-	-	-	-	-	-	-	0	0.11
**DIXDC1 chr11:111797698**	-	-	-	-	-	-	0.56	-	0.12	0.11
**SMUG1 chr12:54582890**	-	-	-	-	-	-	0.41	(0.16)	0.12	0.11
**SMUG1 chr12:54582889**	-	(0.17)	-	-	-	-	0.42	-	0.12	0.11
**CDC20 chr1:43824529**	-	-	-	0.24	-	(0.026)	0.78	0.2	0.25	0.11
**SECISBP2 chr9:91933357**	-	-	-	-	0.8	-	-	-	0.12	0.11
**ARHGEF18 chr19:7459940**	0.21	-	0.88	-	0.21	0.23	0.47	0.63	0.75	0.11
**ARHGEF18 chr19:7459941**	-	-	0.83	-	-	-	-	0.3	0.25	0.11
**WDR82 chr3:52322052**	-	-	-	-	-	-	-	-	0	0.11
**SYNJ1 chr21:34100374**	-	-	-	-	-	-	-	0.52	0.12	0.11
**POLR2D chr2:128615744**	(0.033)	-	-	0.55	-	-	-	-	0.12	0.11
**DHX16 chr6:30640796**	-	-	-	-	-	-	-	-	0	0.11
**MAP1S chr19:17830242**	-	-	0.67	(0.029)	-	-	-	(0.023)	0.12	0.11
**PRKAG1 chr12:49412648**	-	-	-	-	-	(0.069)	-	(0.04)	0	0.11
**Total no. of mutations [c]**	24961	64326	85537	88427	116673	119549	224931	267306		
**Total no. of promoter hotspot mutations [d]**	2	2	5	6	5	3	9	7		
**NOTCH1 [e]**	1	1	3	1	0	1	3	1		
**NOTCH2**	0	2	2	1	2	1	4	2		
**CDKN2A**	0	1	0	0	1	0	1	1		
**TP53**	0	0	1	1	1	0	1	2		
**Total no. of driver mutations**	1	4	6	3	4	2	9	6		
