# Supplementary material for: Recurrent promoter mutations in melanoma are defined by an extended context-specific mutational signature
Source: PLoS Genet. 2017 May 10;13(5):e1006773. doi: 10.1371/journal.pgen.1006773 (PMC5443578; doi:10.1371/journal.pgen.1006773)
Supplement: S5 Table — aMedian number of somatic mutations per tumor derived from whole-genome sequencing data. cSCC counts from Zheng et al. [18]. All other counts from Fredriksson et al. [10]. bUV-radiation as the mutational process driving tumor development. cPresence of mutational signatures 2, 7, 11 or 13 [8], all of which have elevated ratios of C to T mutations in CCT or TCT contexts, which allow for mutations of melanoma promoter hotspot sites. dPresence of TERT promoter mutations[10]. ePresence of melanoma promoter hotspot mutations. fData not available. (PDF) [file pgen.1006773.s008.pdf]

Cancer	Mutation load[a]	UV radiation[b]	Mutational signatures[c]	TERT promoter mutations[d]	Melanoma promoter hotspots[e]
**Prostate, PRAD**	1361				
**Thyroid, THCA**	2055		2	+	
**Low-grade glioma, LGG**	2873			+	
**Kidney (chrom.), KICH**	5147				
**Breast, BRCA**	6194		2, 13		
**Kidney (clear), KIRC**	7234				
**Head & neck, HNSC**	7324		2, 7		
**Uterus, UCEC**	8352		2		
**Glioblastoma, GBM**	9240		11	+	
**Bladder, BLCA**	16011		2, 13	+	
**Lung (adeno), LUAD**	18942		2	+	
**Colorectal, CRC**	21994				
**Lung (squamous), LUSC**	37741		2		
**Melanoma, SKCM**	52663	+	7, 11	+	+
**Skin, cSCC**	102550	+	-[f]	-[f]	+
