# Supplementary material for: Recurrent promoter mutations in melanoma are defined by an extended context-specific mutational signature
Source: PLoS Genet. 2017 May 10;13(5):e1006773. doi: 10.1371/journal.pgen.1006773 (PMC5443578; doi:10.1371/journal.pgen.1006773)
Supplement: S6 Table — Motif search in the JASPAR database using the tool TOMTOM[43]. The motif CTTCCG was compared with motifs in the databases for human transcription factors (HOCOMOCOv10). (PDF) [file pgen.1006773.s009.pdf]

Rank	Name	p-value	E-value	q-value	Overlap	Offset	Orientation
1	ETV6	5.06e-05	3.25e-02	4.07e-02	6	2	Reverse Complement
2	GABPA	6.75e-05	4.33e-02	4.07e-02	6	3	
3	ELK1	1.14e-04	7.33e-02	4.07e-02	6	1	Reverse Complement
4	ELK4	1.28e-04	8.22e-02	4.07e-02	6	3	
5	GABP1	1.80e-04	1.16e-01	4.58e-02	6	3	Reverse Complement
6	ELF2	2.56e-04	1.64e-01	5.43e-02	6	6	Reverse Complement
7	ELF1	3.96e-04	2.54e-01	7.18e-02	6	3	Reverse Complement
8	ERG	5.63e-04	3.61e-01	8.93e-02	6	3	Reverse Complement
9	EHF	1.15e-03	7.35e-01	1.62e-01	6	2	Reverse Complement
10	ETV1	1.52e-03	9.75e-01	1.81e-01	6	10	Reverse Complement
11	ETS1	1.57e-03	1.01e+00	1.81e-01	6	1	Reverse Complement
12	FLI1	1.88e-03	1.21e+00	1.99e-01	6	5	Reverse Complement
13	ETS2	2.23e-03	1.43e+00	2.03e-01	6	3	Reverse Complement
14	STAT3	2.23e-03	1.43e+00	2.03e-01	6	0	Reverse Complement
15	ETV4	2.42e-03	1.55e+00	2.05e-01	6	1	Reverse Complement
16	ELK3	3.70e-03	2.37e+00	2.94e-01	6	3	Reverse Complement
17	SPIB	4.26e-03	2.73e+00	3.04e-01	6	0	Reverse Complement
18	SPDEF	4.32e-03	2.77e+00	3.04e-01	6	4	Reverse Complement
19	ETV5	4.87e-03	3.12e+00	3.22e-01	6	4	Reverse Complement
20	STAT4	5.08e-03	3.26e+00	3.22e-01	6	0	Reverse Complement
21	ELF5	9.79e-03	6.28e+00	5.92e-01	6	2	Reverse Complement
22	ETV7	1.17e-02	7.52e+00	6.77e-01	6	6	Reverse Complement
