# Supplementary material for: Recurrent promoter mutations in melanoma are defined by an extended context-specific mutational signature
Source: PLoS Genet. 2017 May 10;13(5):e1006773. doi: 10.1371/journal.pgen.1006773 (PMC5443578; doi:10.1371/journal.pgen.1006773)
Supplement: S8 Table — (PDF) [file pgen.1006773.s011.pdf]

Gene	Position	Forward primer 5' - 3'	Reverse primer 5' - 3'	Amplicon length
RPL13A	chr19:49990642-49990705	CCCACGTGATCTCTCGCC	GCCCTCTTCCGAATGTCCAA	83
DPH3	chr3:16306469-16306523	TCGGCATCATCAGGTCGC	GGGCGGAGTTGCGTCA	70

Universal fwd primer	GGACACTCTTTCCCTACACGACGCTCTTCCGATCTNNNNNNNNNNNNATGGGAAAGAGTGTCC-fwd target primer
Universal Rev primer	GTGACTGGAGTTCAGACGTGTGCTCTTCCGATCT-rev target primer
Illumina fwd primer	AATGATACGGCGACCACCGAGATCTACACTCTTTCCCTACACGACGCTCTTCCGATCT
Illumina rev primer with index	CAAGCAGAAGACGGCATACGAGATNNNNNNGTGACTGGAGTTCAGACGTGTGCTCTTCCGATCT
Index primer	GATCGGAAGAGCACACGTCTGAACTCCAGTCAC
Sequencing primer	ACACTCTTTCCCTACACGACGCTCTTCCGATCT
